# How rapid advances in imaging are defining the future of precision radiation oncology

**DOI:** 10.1038/s41416-019-0412-y

**Published:** 2019-03-26

**Authors:** Laura Beaton, Steve Bandula, Mark N. Gaze, Ricky A. Sharma

**Affiliations:** 1 Cancer Institute, University College London, London, UK; 2 NIHR University College London Hospitals Biomedical Research Centre, UCL Cancer Institute, University College London, London, UK

**Keywords:** Radiotherapy, Cancer imaging

## Abstract

Imaging has an essential role in the planning and delivery of radiotherapy. Recent advances in imaging have led to the development of advanced radiotherapy techniques—including image-guided radiotherapy, intensity-modulated radiotherapy, stereotactic body radiotherapy and proton beam therapy. The optimal use of imaging might enable higher doses of radiation to be delivered to the tumour, while sparing normal surrounding tissues. In this article, we review how the integration of existing and novel forms of computed tomography, magnetic resonance imaging and positron emission tomography have transformed tumour delineation in the radiotherapy planning process, and how these advances have the potential to allow a more individualised approach to the cancer therapy. Recent data suggest that imaging biomarkers that assess underlying tumour heterogeneity can identify areas within a tumour that are at higher risk of radio-resistance, and therefore potentially allow for biologically focussed dose escalation. The rapidly evolving concept of adaptive radiotherapy, including artificial intelligence, requires imaging during treatment to be used to modify radiotherapy on a daily basis. These advances have the potential to improve clinical outcomes and reduce radiation-related long-term toxicities. We outline how recent technological advances in both imaging and radiotherapy delivery can be combined to shape the future of precision radiation oncology.

## Introduction

At present, around 40% of cancer cures involve the use of radiotherapy, either as a single modality or in combination with other treatments such as surgery or chemotherapy.^1,2^ Radiotherapy is also highly effective for palliation and for symptom control in patients with advanced stage or recurrent cancer.^3,4^ In addition, an increasing body of evidence now supports the use of high-dose radiotherapy in the treatment of oligometastatic disease; radiation is delivered to a small number of metastases, with studies showing improved local control rates for several tumour sites.^5–7^

Over the past decade, a number of significant technical developments, such as image-guided radiotherapy (IGRT), intensity-modulated radiotherapy (IMRT), stereotactic body radiotherapy (SBRT) and proton beam therapy (PBT), have enabled the concept of ‘precision radiotherapy’ to be advanced rapidly.^8^ Such advanced radiotherapy techniques facilitate greater conformity, reduced planning margins and the delivery of an increased dose to the tumour, potentially resulting in improved patient outcomes.^5,6,9^ The delivery of highly conformal, high-dose radiotherapy is, however, challenging due to uncertainties in the accuracy of imaging, treatment planning, treatment delivery and even changes in tumour size during treatment. The integration of imaging technology into modern radiotherapy treatment machines has led to an increase in the precision and accuracy of radiation delivery.^10^ Imaging throughout a course of radiation treatment enables anatomical changes to be detected, and radiation plans to be adapted accordingly. Indeed, adaptive radiotherapy, whereby an individual plan can be created for each treatment fraction depending on the precise positioning, and shape, of the tumour at the time of imaging, is a new goal for radiation oncology. For this goal to become a realistic possibility, advanced imaging and the integration of automated segmentation processes for the delineation of target volumes and surrounding structures need to be developed.

The first step in being able to deliver precision radiotherapy is accurate target delineation during the radiotherapy planning process. Advanced multi-modality diagnostic imaging with computed tomography (CT), high-resolution magnetic resonance imaging (MRI) and 18-F-fluorodeoxyglucose positron emission tomography (FDG-PET)/CT imaging now forms part of the routine staging process for a number of tumour types.^11–14^ The development and application of new functional imaging techniques might offer a greater understanding of individual tumour biology and could help to identify potential areas of radio-resistance within a tumour that require radiation dose escalation.

In this review, we begin by outlining the target delineation process before providing an overview of recent advances in technology relevant to biological target volume definition, IGRT, adaptive radiotherapy and the technologies under development that will define the future of precision radiation oncology.

## Target delineation

The more conformal and precise radiation delivery becomes, the more vital it is to accurately identify the tumour, its local extension and the exact location of surrounding critical structures: organs at risk (OARs). Inaccuracies in tumour and OAR delineation are recognised as a common source of radiation therapy error.^15^ Errors in delineation can ultimately lead to an insufficient dose being delivered to the tumour, leading to reduced tumour control, or too high a dose being delivered to nearby OARs, resulting in increased toxicity for the patient. Imaging thus serves a vital role in defining the targets for radiation therapy.

The radiotherapy planning process typically starts with the acquisition of a CT scan with the patient immobilised in the optimal radiotherapy treatment position. CT scans are the standard imaging modality for radiotherapy planning, as they provide a three-dimensional (3D) view of the tumour as well as data regarding electron density, which are required for dose calculations.^16^ The macroscopic, radiologically measurable tumour is delineated as the gross tumour volume (GTV). Tumour delineation from CT imaging can be challenging, especially for soft tissues. To improve the accuracy of GTV delineation, algorithms have been developed that allow co-registration of diagnostic images, particularly those derived from MRI and FDG-PET, with the primary simulation CT scan.^17,18^ MRI provides higher resolution and greater soft-tissue contrast in pelvic tumours when compared to CT imaging, while FDG-PET provides more accurate tumour delineation in head and neck, lung, lymphoma and oesophageal cancers.^19–22^ Registration can be relatively simple (rigid), where the transformation preserves the distance between all points, or more complex (non-rigid or deformable), which involves a significantly greater number of degrees of freedom and allows for distortion between two image sets. Such co-registration or ‘fusion’ of images allows for the GTV to be contoured on to the planning CT dataset, while using information from the diagnostic images to ensure accurate tumour delineation. The development of MR compatible devices—devices that are safe and unaffected by the MR environment—also means that MRI can now be performed with the patient in the radiotherapy planning position.^23^ This approach avoids the need for complex registration algorithms, and helps to minimise inaccuracy that may occur during the image fusion process.

A second volume, the clinical target volume (CTV), adds a margin to the GTV to cover neighbouring sites that are at risk of harbouring microscopic disease, and that are important to target to achieve cure.^24–26^ Delineation of the CTV is based on anatomical/spatial information gained from cross-sectional imaging. However, this information might be an oversimplification, as it does not reflect the underlying complexity of the tumour; instead, current practice is to deliver a relatively homogeneous radiation dose to the entire CTV.^27^ Information on the risk and extent of tumour spread in expanding GTV to CTV is based on historical practice rather than the extent of tumour quantified on imaging for an individual patient.^28^

**Fig. 1:**
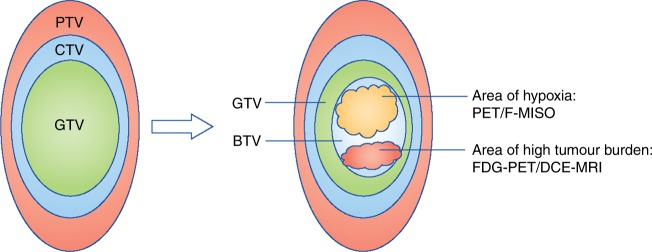
**Target volume definitions and the concept of the biological target volume.** At present, target volume (tumour) delineation is characterised by the concepts of gross tumour volume (GTV), clinical target volume (CTV) and planning target volumes (PTV). Information from functional imaging may provide additional information for defining the ‘biological target volume’ (BTV)—a subvolume of the tumour that may indicate underlying radio-resistance. For example, hypoxic areas may be derived from PET-^18^F-misonidazole (F-MISO) imaging and high tumour burden from 18-F-fluorodeoxyglucose positron emission tomography (FDG-PET)/CT or dynamic contract enhanced magnetic resonance imaging (DCE-MRI). A higher dose of radiation can then be delivered to the BTV (rather that the whole tumour) in a process known as dose painting (image adapted from Ling et al.^29^)

The planning target volume (PTV) is a geometrical concept that allows for uncertainty in planning or treatment delivery. It takes into account the internal motion of the tumour during treatment (internal target volume (ITV)), in addition to random and systematic variations that occur in patient set-up on a daily basis. These variations can include changes in the tumour position and shape due to tumour regression or growth, bladder filling or rectal distension, as well as unpredictable changes arising from a change in the patient position or set-up methodology between each delivered fraction. The PTV is therefore the recommended parameter to ensure that an adequate dose of radiotherapy will actually be delivered to all parts of the CTV (Fig. 1).^26^

**Fig. 2:**
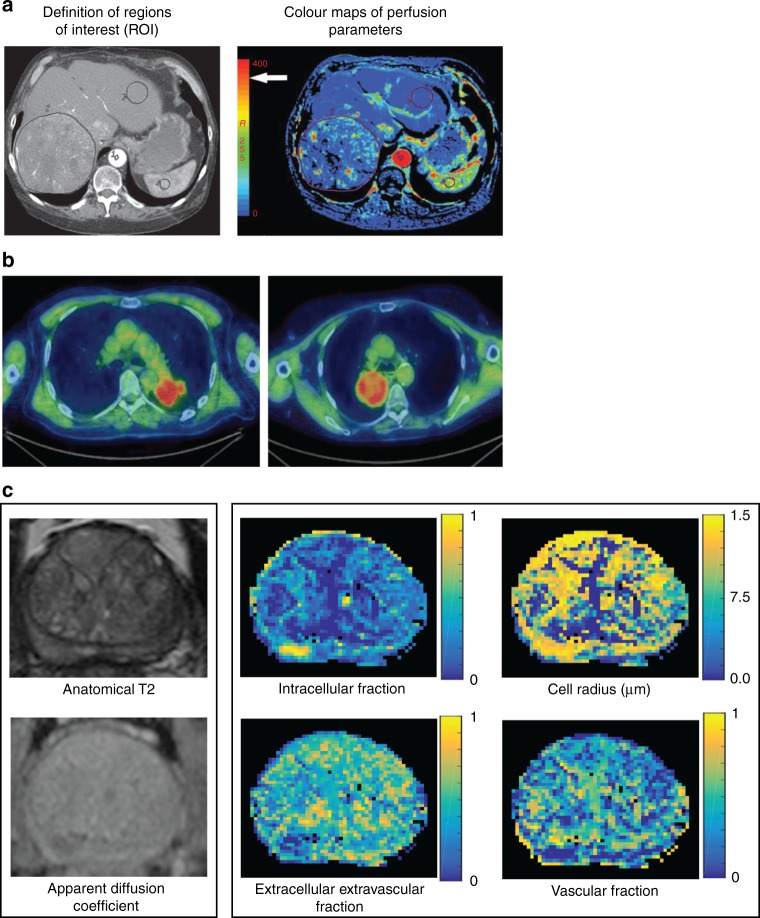
**Examples of functional imaging techniques.** **a** Functional CT technique for measuring perfusion parameters (in tumours and normal liver). Perfusion CT images from a patient with a large liver metastasis from colorectal cancer. (Left panel) Aorta (marked 1), liver tumour (marked 2) and normal tissue (marked 3) delineated as regions of interest (ROIs); (right panel) Colour maps of perfusion parameters within ROIs. Red indicates higher blood flow. **b** 18-F‐fluoromisonidazole uptake in advanced stage non‐small cell lung cancer.^64^ Two examples of F-MISO PET/CT images on an SUV scale 0–3 for two different patients with non-small cell lung cancer. Images courtesy of Geoff Higgins, Oxford Institute for Radiation Oncology. **c** ADC and VERDICT magnetic resonance images from a patient with prostate cancer. MRI images from a patient with prostate cancer (biopsy confirmed). (Left panel) Axial T2-weighted image and ADC map from standard mpMRI; and (right panel), vascular Extracellular Restricted Diffusion for Cytometry in Tumours (VERDICT) volume fraction maps of the intracellular, the extracellular-extravascular and the vascular components, and the cell radius index map.^48^ Images courtesy of Laura Panagiotaki, University College London. ADC apparent diffusion coefficient, mpMRI multi-parametric magnetic resonance imaging, VERDICT Vascular Extracellular Restricted Diffusion for Cytometry in Tumours

A greater understanding of tumour biology, and a recognition of the need to consider tumour heterogeneity, has led to attempts to incorporate information from new functional imaging techniques into treatment planning. Incorporating both metabolic and molecular imaging techniques into the radiotherapy planning process fosters the concept of a ‘biological target volume’ (BTV) (Fig. 1). Delineating a BTV would take into account the metabolic, biochemical, physiological and functional changes within a tumour, and has the potential to advance tumour delineation away from being a purely anatomical/spatial exercise.^29^ FDG-PET imaging is currently the most widely used functional imaging technique that could facilitate BTV delineation, although a number of other novel functional imaging techniques are in development (Fig. 2). Functional imaging can map tumour characteristics, such as hypoxia, vascularity and cellular proliferation, that are known to impact the outcome of radiotherapy. This has led the search to identify reliable ‘imaging biomarkers’—qualitative or quantitative measurements derived from imaging modalities that can map spatial heterogeneity within the tumour.^30^ The incorporation of these imaging biomarkers into the radiation planning process could therefore highlight areas at risk of radio-resistance, and allow for focused biological dose escalation.^31^

## Advances in CT imaging

CT is the most widely used imaging modality for cancer staging and response assessment. A standard diagnostic CT scanner comprises of an X-ray source and a detector, mounted on a rotational gantry that moves around the patient. Computing algorithms are then used to create cross-sectional images from the mathematical reconstruction of the measured X-ray intensities from the detector. New reconstruction algorithms, particularly iterative reconstruction, and increased detector sensitivity have facilitated a significant reduction in exposure to ionising radiation, and continuous improvements in data processing (e.g. noise and artefact correction) have enabled high-quality CT imaging for cancer. These developments have not only driven an improvement in anatomical image quality but have spawned a number of quantitative CT techniques that are better able to characterise tissue and describe function. Imaging biomarkers derived from techniques such as perfusion imaging and dual-energy CT could improve target definition and form the basis for more individualised cancer therapy.

Perfusion CT, or dynamic contrast-enhanced CT (DCE-CT), imaging employs standard low molecular weight iodinated contrast agents. When administered as an intravenous bolus, tissue concentration is dependent on vascular flow and interstitial accumulation, resulting in differential attenuation on CT imaging, before recirculation and clearance of the agent by the kidneys. Neovascularisation and hyperpermeability within the tumour vasculature results in a concentration curve that differs from that of healthy tissue, with a more rapid initial uptake of contrast, high peak enhancement and rapid washout.^32^ Qualitative and semi-quantitative evaluation and mapping of these data can improve tumour detection and differentiation of tumour tissue from healthy or treated tissues.^33^ More complex kinetic perfusion models can derive functional physiological parameters such as blood flow, blood volume, mean transit time, extracellular-extravascular volume fraction, and permeability surface area product. Blood flow reflects the delivery of oxygen and other nutrients to tumour tissue, and is an indirect measure of hypoxia and angiogenesis, whereas the permeability surface area product reflects the leakiness of tissue vasculature, and interstitial pressure. Neovascularisation, hypoxia and reduced contrast extraction are parameters associated with tumour aggressiveness and treatment resistance, and could be specifically targeted.

Tumour perfusion rate has been assessed as an imaging biomarker in patients with head-and-neck squamous cell carcinoma (HNSCC) treated with radiotherapy and found to be an independent predictor of local control of the tumour—patients with lower perfusion rates had a significantly higher local failure rates.^34^ Studies have also investigated changes in tumour perfusion following treatment with systemic therapy.^35,36^ The identification of such biomarkers, that represent areas at risk of radio-resistance, may therefore highlight areas within the tumour that may require focal dose escalation.

Contrast resolution, and therefore sensitivity to abnormal tissue (hypo-enhancing or hyper-enhancing), can be improved by using dual-energy CT techniques to produce low-tube-voltage imaging. Dual-energy CT imaging is a technique that uses attenuation data acquired at two different tube voltage settings. Using these data, images at any tube voltage can be reconstructed. At low-tube-voltages the X-ray absorption of iodine is increased relative to water and soft tissues, resulting in increased image contrast. This technique has been shown to increase the conspicuity of hypervascular liver^37^ and hypovascular pancreatic tumours,^38^ and could aid with tumour delineation in the future.

## Advances in MRI

Standard MRI relies on the magnetic properties of hydrogen protons within a tissue to generate image contrast. The past decade has seen the development of several new quantitative MRI techniques that have expanded the use of these properties to start to probe the underlying tissue microstructure.

### Diffusion-weighted MRI

Diffusion-weighted MRI has already been widely adopted in cancer imaging due to its ability to enhance soft-tissue contrast. Diffusion-weighted imaging (DWI) adds further sensitivity to the detection of the intrinsic random thermal movement of hydrogen protons within water molecules that occurs as part of the natural (Brownian) diffusion process of water. Sensitisation to this motion is achieved with a pair of equal and opposing magnetic gradients, applied in succession, such that any net displacement of water dipoles can be attributed to diffusion along the orientation of the field gradients. Measured DWI signals from water movement within tissues thus reflect properties such as cellular density, intracellular structure, membrane permeability, interstitial volume and blood flow.

Tumour growth, resulting in areas of increased cellularity, tends to cause restriction of water motion, which can be quantified by the apparent diffusion coefficient (ADC). A change in tumour cellularity, perfusion and cellular necrosis following treatment is reflected in a change in the ADC value, and the potential utility of DWI in tumour detection, grading and assessment of treatment response has been demonstrated in a range of tumour types.^39,40^ ADC values have been assessed in patients with HNSCC treated with chemoradiation. Significantly lower pre-treatment ADC values were found in complete responders when compared to partial responders, suggesting that this biomarker could be used to predict response to treatment and may even allow for dose-modification.^41^ Technological advances have allowed quantitative functional assessment with MRI to be broadened into whole body acquisitions (WB-MR-DWI) with accurate staging of cancers including lymphoma^42^ and myeloma.^43^

**Fig. 3:**
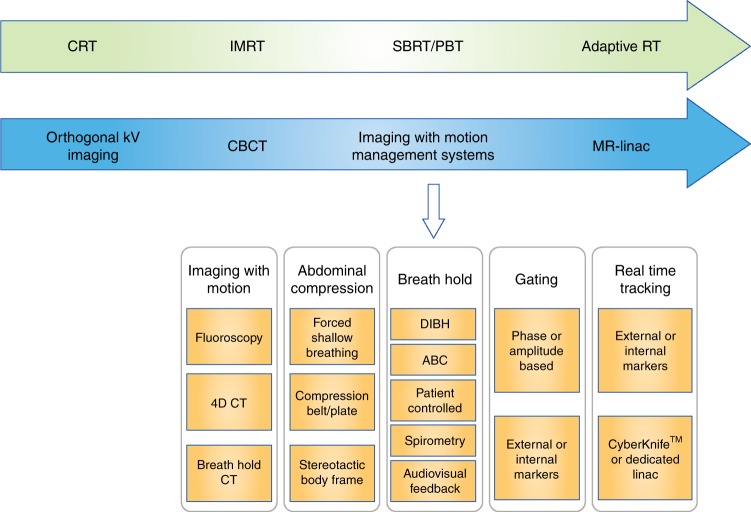
**Overview of advances in radiotherapy techniques and image-guided radiotherapy (IGRT) over time.** Recent advances in imaging techniques have enabled tumours to be more accurately delineated for radiotherapy treatment and for image guidance during therapy. The advent of CBCT and motion management systems has allowed radiotherapy to become more highly conformal, and high ablative doses can be safely delivered with sparing of surrounding normal tissues. The development of the MR Linac takes this one step further by offering the prospect of real-time imaging using MRI to permit daily adaptive radiotherapy. CRT conformal radiotherapy; IMRT intensity-modulated radiotherapy; SBRT stereotactic body radiotherapy; PBT proton beam therapy; CBCT cone beam CT**;** DIBH deep-inspiration breath hold; ABC active-breathing control

ADC values are derived from the application of a simple (monoexponential) model to the DWI-MRI. This model is based on the approximation of the diffusion of water molecules within a tissue. More complex models that divide the diffusion signal into pseudo-diffusion (like perfusion) and true molecular diffusion can also be applied (intravoxel incoherent motion; IVIM).^44^ This approach allows separation of the diffusion and perfusion signal component of the water molecules, and could improve tissue characterisation such that tumours might be distinguished by pathological grade.^45,46^ However, these existing DWI models lack specificity for various elements of the tissue microstructure, such as cell size, shape, intracellular structure, and perfusion effects. An improved framework for describing diffusion behaviour was proposed by Panagiotaki et al.^47^ This Vascular, Extracellular, and Restricted Diffusion for Cytometry in Tumours (VERDICT) method uses a three-compartment model to specifically account for intracellular, interstitial and intravascular water and map descriptors, which form key components of histopathological tumour assessment (Fig. 3). VERDICT has demonstrated an early potential to provide clinically useful microstructural information in patients with prostate cancer^48^ and bone metastases.^49^

### Dynamic contrast-enhanced-MRI

Dynamic contrast-enhanced-MRI (DCE-MRI) is a quantitative MRI technique that allows investigation of tissue microvascular structure and function by evaluating the pharmacokinetics of an extracellular low molecular weight contrast agent (e.g. gadolinum-based agents) as it passes through the tumour vasculature. The lack of ionising radiation allows temporal sampling frequency to be limited only by sequence acquisition time, and the technique is therefore attractive for repeated interval assessments. DCE-MRI is sensitive to alterations in vascular volume, blood flow and vascular permeability, and its role in tumour assessment has been extensively investigated^50–52^ The technique has become the backbone of some MRI protocols for breast examinations and a sensitive imaging test for breast cancer detection^53^ with qualitative analysis of contrast kinetics. DCE-MRI is, however, complicated by the non-linear relationship between contrast agent concentration and signal intensity, and its dependence on scan parameters such as flip angle, repetition time and pre-contrast signal. Furthermore, quantitation can be complex, and measurement variation across centres remains an issue, which has limited the clinical implementation of DCE-MRI in cancer imaging.^54^ Some of these limitations could potentially be overcome by combining more than one type of MRI imaging in each patient to personalise treatment planning and assessment.^55,56^

### Magnetic resonance spectroscopy

Magnetic resonance spectroscopy (MRS) is a technique based on the detection of radiofrequency signals generated by specific endogenous nuclei (such as ^1^H, ^31^P, ^13^C and ^19^F). MRS allows the analysis and quantification of metabolites present in tissue, such as choline, creatine and myoinositol, which could act as tumour biomarkers. MRS has shown promise in the diagnosis and monitoring of tumours in the brain, prostate and breast.^57–59^ The technique is, however, technically complex, offering lower signal strength and sensitivity in comparison to other MRI techniques, which has limited its clinical applicability.

### Blood oxygen level-dependent MR imaging

Blood oxygen level-dependent (BOLD) MRI is a non-invasive functional imaging method that exploits endogenous contrast.^60^ Blood can confer intrinsic contrast dependent on the degree of oxygenation and its effect on the T2 signal. Deoxyhaemaglobin is paramagnetic (that is, it has one or more unpaired electrons, which results in a strong electronic magnetic moment), and causes increased signal loss on T2*-weighted imaging compared with diamagnetic oxyhaemaglobin (in which only paired electrons are present). This fundamental difference in the paramagnetic properties of oxygenated and deoxygenated blood means that BOLD MR imaging is able to detect changes in oxygenation within a tissue. Although BOLD MR methods have mainly been used to map functional activity in the brain, they have also been used to help evaluate tumours^61^ and interventions designed to disrupt tumour oxygenation.^62^ In the context of radiotherapy planning, BOLD-MRI may therefore enhance treatment plans by improving the delineation of critical neural structures that must be spared from high radiation doses. Furthermore, BOLD-MRI may provide a non-invasive method of imaging tumour hypoxia, which could identify tumours (or even areas within a tumour) at risk of radio-resistance, and could predict response to treatment.

### Chemical exchange saturation transfer MRI

Molecular information can be extracted by MRI methods that exploit the difference in magnetic properties between tissues of different composition. Signal can be modulated by cross interaction between mobile water and relatively immobilised protons within macromolecules. By using selective radiofrequency pulses to excite the magnetisation of exchangeable protons (within, for example, amides and hydroxyls) and decrease the associated water MRI signal because of proton magnetic interaction, Chemical Exchange Saturation Transfer MRI is able to sensitise the acquisition to smaller macromolecules. Signal from even low concentrations of metabolites is magnified and each can be mapped separately.^63^

## Advances in positron emission tomography

Positron emission tomography (PET) is a functional imaging technique involving the use of radioactive tracers—positron emitters attached to targeted biologically active molecules. 3D images of tracer concentration within the body are then constructed to assess the distribution of the targeted molecule in vivo. FDG, an analogue of glucose, is the most frequently used tracer, as FDG avidity reflects a number of key biological processes likely to be relevant to radiotherapy response, including receptor-mediated glucose uptake, cellular respiration, hypoxia and perfusion.^31^ Other PET tracers have since been developed, including fluorine-18 fluoromisonidazole (F-MISO),^64^ which binds to hypoxic areas of the tumour, and 3′-deoxy-3′-fluorothymidine (FLT), which is a measure of tumour proliferation. PET-CT and PET-MRI with ^18^F-fluoroethylcholine have also been shown to be of value in the diagnosis and response assessment of paediatric brain tumours.^65,66^

^124^I is a positron-emitting isotope of iodine, and ^124^I-labelled metaiodobenzylguanidine (mIBG) can be used in the assessment of neuroblastoma by combined PET-CT or PET-MRI, in place of ^123^I-mIBG scintigraphy and single photon emission computed tomography (SPECT)-CT, to identify diseased areas with greater resolution. The longer half-life of ^124^I-mIBG allows serial scans to be performed over several days to track the biokinetics of the radiopharmaceutical. This might have predictive value for dosimetry prior to ^131^I-mIBG molecular radiotherapy.^67^

## Role of functional imaging in biological target volume delineation

Although functional imaging techniques have the potential to provide important prognostic information on a tumour prior to radiotherapy treatment, translating this information into improving precision radiotherapy remains a challenge. The aim is that functional imaging techniques would identify subvolumes of a tumour representing areas of radio-resistance–the biological target volume—and that these areas would then receive an extra dose of radiation with high precision without increasing the dose to the whole tumour.

A number of radiotherapy planning studies have assessed the role of hypoxia to target biological dose escalation. These have mainly involved PET-based imaging, including F-MISO, and have focused on head and neck cancers.^68,69^ Early clinical trials have also used tumour burden, based on FDG-PET and DCE-MRI imaging, to enable focused escalation.^70–72^ These trials show that focused dose escalation using functional imaging is feasible, but large prospective clinical trials are required to establish the benefit for tumour control and toxicity. Although moving from a GTV to a BTV is the ultimate aim in being able to deliver precision biologically-directed radiotherapy, a number of challenges still need to be overcome before this approach becomes standard clinical practice. At present, there is no single imaging modality that reflects all underlying mechanisms of radio-resistance, and studies have shown that it may be difficult to establish the spatial relationships between imaging techniques.^73^ Imaging biomarker prioritisation will therefore need to be established before functional imaging can be reliably used for dose escalation.

## Image guidance and advances in radiotherapy delivery

The delivery of radiotherapy has changed significantly over the past two decades. The introduction of 3D imaging has moved radiation delivery away from simple treatment fields to a more complex approach using 3D conformal radiotherapy (CRT). This was followed by the introduction of IMRT, an advanced form of CRT that optimises the delivery of radiation to irregularly shaped objects.^16^ While traditional radiation treatments deliver a consistent intensity across the treatment field, IMRT uses multiple beams to deliver the dose, with a high degree of intensity modulation across each beam.^74,75^ Advanced computation, including inverse planning—an optimisation process which begins with specification of the desired dose to the tumour targets and dose limits to critical structures—is required to decide how to alter the intensity of the beam in order to make the treatment most conformal. IMRT can therefore allocate different dose targets for treatment to achieve optimal dose distributions.^75^ This technique enables dose escalation to the tumour target or, potentially, to defined ‘high risk areas’ within the target (creating an inhomogeneous dose across the tumour), while sparing dose to the surrounding normal tissue.

### Image guidance for state-of-the-art radiotherapy

Dose escalation using IMRT has been shown to improve outcomes at a number of tumour sites, without increasing toxicity.^76–79^ Intensity-modulated arc therapy (IMAT) is a form of IMRT, but in this approach the linear accelerator gantry head rotates in an arc around the patient;;^80^ it shows improved conformity and reduced treatment times.^81^ Optimal IMRT or IMAT delivery does, however, require more advanced image guidance than 3D CRT does. For 3D CRT, patients are aligned for daily treatment with the use of external skin marks (tattoos) and correct positioning is verified on radiographs by matching to anatomical landmarks.^82^ With the greater dose conformality provided by IMRT or IMAT, however, there is a greater need to reliably localise the target at the time of treatment.

The development of on-board imaging systems means that linear accelerators are now capable of performing cone beam CT (CBCT) scans prior to treatment, which increases the certainty of tumour location at the time of treatment. Imaging with CBCT can identify daily changes in tumour position due, for example, in the case of pelvic tumours, to changes in bladder and bowel filling. Localising the tumour can be enhanced further by the implantation of fiducial markers, either within or near to the tumour, prior to the start of treatment.^75^ As such, CBCT imaging prior to treatment enables the safe delivery of radiotherapy by IMRT and IMAT approaches.

Traditionally, radiation treatments are fractionated into daily doses of 1.8–2.0 Gy to maximise the therapeutic ratio between the dose to the tumour and to normal tissue, and this approach applies to CRT and IMRT techniques. SBRT is an alternative method that accurately delivers a high dose over one to five fractions to an extracranial target.^83,84^ The doses delivered in SBRT are therefore ‘ablative’ and, accordingly, the safe delivery of such large doses per fraction necessitates effective patient immobilisation, precise target localisation, accurate treatment delivery and daily image guidance to allow very steep isodose gradients between target volumes and surrounding normal tissues.^83,85^ The excellent results achieved with ablative doses suggest that the anti-tumour effects of SBRT are different to those induced by conventional fractionation. Endothelial cell damage, enhanced anti-tumour immunity and a lack of time for reoxygenation and repopulation are possible underlying mechanisms for the increased efficacy of SBRT.^86^

Recent advances in IGRT allow for patient movement due to respiration to be measured and accounted for during treatment.^83,85^ These advances include ‘motion-encompassing methods’ (in which motion will be present during radiotherapy), ‘forced shallow breathing’ techniques, ‘breath hold’ techniques, ‘respiratory gating’ techniques and ‘respiration synchronised’ techniques.^87^ The advent of four-dimensional CT (4D-CT) imaging, or respiratory-correlated CT imaging, has allowed high-quality CT data to be obtained at the radiotherapy planning phase in the presence of respiratory motion. 4D-CT data can then be analysed to assess the mean tumour position and tumour range for treatment planning, in addition to the relationship of the tumour position to phases of respiration.^87^ Tumour motion throughout the breathing cycle can then be accounted for when the radiation plan is created, delivered and verified. Abdominal compression devices can restrict respiratory motion and enforce shallow breathing patterns. Alternatively, motion can be arrested through voluntary or device-assisted schemes and radiation delivered only when the patient holds his or her breath (using ‘breath hold’ techniques). These schemes include deep-inspiration breath hold and active-breathing control systems. Treatment machines that interrupt radiation delivery when the tumour is out of position (gating techniques) can also be used. This approach involves verification of the patient’s anatomy with in-room imaging, and the use of either an external marker of respiration or implanted fiducial markers that move with the tumour.^10,87^ Finally, ‘respiratory synchronised’ techniques enable the radiation beam to ‘track’ the tumour during treatment, by either movement of the linear accelerator, or movement of the patient. Dedicated stereotactic radiotherapy machines capable of compensating for tumour motion in this way are available. Cyberknife^®^ (Accuray, Sunnyvale, California, USA) is a dedicated robotic delivery system that can deliver SBRT with real-time tracking. This technique can allow radiation delivery with an accuracy of 0.5 mm.^75^

### Proton beam therapy

All of the treatment techniques discussed so far have utilised megavoltage photons to deliver radiotherapy. Charged particle therapy, such as proton beam radiotherapy (PBT), has gained particular interest as an alternative to photons. Protons deposit little energy as they interact with matter, until they reach the end of their range (which depends on their initial energy). This gives rise to a low dose on entering the tissue, which reaches a relatively flat dose plateau before rising sharply to a maximum, the Bragg peak, and then falling to zero at the end of the beam range. This unique characteristic dose release pattern has led to the use of PBT in paediatric cancers (in order to reduce late effects and second primary malignancies), and in adults to provide an effective dose to tumours that are localised adjacent to structures sensitive to the effects of radiation, such as the brainstem and spinal cord.^88,89^ One potential limitation of PBT is that the range of a proton is subject to considerable uncertainties caused by imaging, patient set-up, beam delivery and dose calculation.^90^ Proton ranges are highly sensitive to the composition of the media through which the beam traverses, and so are highly sensitive to variations in patient positioning.^91,92^ As the range of a proton beam varies as a function of the relative stopping power (RSP) of the absorbing material, there is a degree of range uncertainty due to the conversion of Hounsfield units (HU) in the planning CT scan to RSP. This uncertainty is ~2% for soft tissue, and as high as 5% for lung, fat, and bone; an average value of 3.5% is often assumed for clinical practice.^93–95^ To reduce this range uncertainty, there is a developing interest in using dual-energy CT imaging as opposed to standard single-energy CT imaging. Dual-energy CT imaging provides greater information on the elemental composition of the absorbing material, as the attenuation of the material is known at two energies; recent studies have suggested that dual-energy CT imaging could improve the accuracy of the predicted RSP, and therefore the estimation of proton range, in the order of 0.1–2.1%.^96,97^

### Molecular radiotherapy

Molecular radiotherapy, sometimes called radionuclide therapy or therapeutic nuclear medicine, is the administration of biologically targeted radiopharmaceuticals which are concentrated and retained in tumour tissue to a significantly greater extent than in normal tissues or OARs, resulting in the sustained delivery of radiation to the cancer at both the primary site and in metastatic deposits. One advantage over external beam therapy is that it is anatomically unconstrained—all tumour cells expressing the physiological uptake mechanism for the radiopharmaceutical in question will be targeted no matter where they are located anatomically. The oldest example is the use of ^131^I-sodium iodide for thyroid remnant ablation and treatment of metastatic differentiated thyroid carcinoma.^98^ Even patients with extensive metastatic disease can be cured using this form of molecular radiotherapy.^99^ Other commonly used treatments are ^131^I-mIBG for childhood neuroblastoma^100^ and ^177^Lu-DOTATATE in the management of neuroendocrine cancers.^101^ A range of studies over the past 30 years have shown that about one in three patients has an objective response to ^131^I-mIBG^102^ and there is level one evidence of a survival benefit of ^177^Lu-DOTATATE.^101^

Accurate imaging is essential for the planning, delivery and evaluation of molecular radiotherapy. Selection of the targeted radiopharmaceutical for treatment is typically based on the use of imaging biomarkers. Semi-quantitative assessment of the uptake of mIBG using ^123^I-mIBG scintigraphy is used for the staging and response assessment of neuroblastoma,^103^ and avid uptake into known sites of disease is an essential prerequisite for ^131^I-mIBG therapy. Similarly in neuroendocrine cancers, the demonstration by PET-CT of the uptake of ^68^Ga-DOTATATE into tumours with a greater standardised uptake value (SUV) than in liver is required before proceeding to ^177^Lu-DOTATATE therapy.^104^

Imaging is essential for tumour dosimetry, which is now mandatory under the European Union Council Directive 2013/59/EURATOM. In relation to all radiotherapeutic exposures, practitioners must ensure that exposures of target volumes are individually planned and their delivery is appropriately verified, taking into account that doses to non-target volumes and tissues must be as low as reasonably practicable and consistent with the intended radiotherapeutic purpose of the exposure. This requires a series (minimum of three, and ideally more) of SPECT-CT scans to allow for quantification of the absorbed dose to the tumour and critical normal organs. Although the administration of a greater activity, proportional to body weight, will result in a higher dose to the whole body, tumour and organs, the precise doses vary depending on the biokinetics of the radiopharmaceutical in individual patients. Personalised molecular radiotherapy can therefore be based on modification of the activity of second and subsequent administrations based on the measured doses achieved following an initial therapy administration, or on measurements taken following the administration of a tracer.

## Adaptive radiotherapy

Radiotherapy practice traditionally involves delivering the original radiotherapy treatment plan to completion, without accounting for anatomical or biological changes that can occur over the course of treatment, which can last up to 7 weeks. However, anatomical changes could occur due to patient weight loss, tumour shrinkage, oedema, inflammation, and normal tissue volume alterations over this time period.^105^ Indeed, recent advances in IGRT, particularly the use of CBCT, have shown that there can be significant volumetric changes to the tumour and OARs during a course of treatment.^106,107^ As radiation can now be delivered with advanced techniques that can create sharp dose gradients at the margins of tumours, even a slight change in anatomy could result in significant dosimetric changes. Imaging throughout the course of a treatment should therefore detect these anatomical changes, and allow for treatment plans to be modified accordingly. At present, imaging is most commonly performed using CBCT (carried out on the treatment unit) either on a daily or weekly basis (depending on the duration and type of treatment schedule and the likelihood of significant movement of the target volume). If significant anatomical changes are detected, then a new radiation plan can be created; this is termed ‘Adaptive Radiotherapy,’ but it is time consuming and can lead to delays in treatment. CBCT imaging, however, does not allow for optimal imaging of tumours and OARs when they are surrounded by soft tissues.^108^ Furthermore, it only allows for the assessment of anatomical, and not biological, changes in response to radiation.

### Role of functional imaging during radiotherapy

A number of studies have therefore evaluated the role of functional and molecular imaging techniques in evaluating the response of the tumour to radiation.^109–112^ Although FDG-PET is the most widely studied imaging modality, the roles of FLT-PET, DCE-MRI and DWI-MRI have also been assessed.^113–116^ Imaging the response of the BTV in this way could be then be used to adapt the radiation plan, and allow for dose escalation. There are, however, several issues in using functional imaging to guide adaptive radiotherapy. First, there will be differences in patient positioning between the planning CT and functional images taken during treatment; as such, a reliance on accurate co-registration algorithms is required. In addition, the optimal timing for assessing functional imaging during a course of radiation is unknown, as is the effect of concurrent chemotherapy or immunotherapy agents on functional imaging (Box 1). Furthermore, as with using functional imaging at the radiotherapy planning stage, tumour delineation presents a challenge—areas of radio-resistance are not defined by a set image intensity level, and as such no threshold can be used to segment out this region. Automated tools that can delineate a tumour based on a threshold SUV of the tracer are difficult to use during a course of radiation as there is a decrease in the ratio of tumour to background uptake.^117^ This is because the SUV of a tumour decreases in response to radiation (indicating a decrease in the number of tumour cells), while no change occurs in the normal background tissue. This results in the automated software not being sensitive enough to distinguish tumour from background and define a volume.^118^

In order to deliver optimal adaptive radiotherapy, images need to be taken throughout the course of the radiation treatment (not just at one time point), anatomical and functional changes need to be assessed, and rapid changes in the radiotherapy plan need to be implemented. These requirements have led to substantial interest in the role of MRI in real time, and online therapy guidance, whereby daily images are used to adapt the radiotherapy treatment plan before each fraction of radiotherapy is delivered.

Box 1 Imaging techniques to assess immunotherapy Tumours have been shown, by imaging, to respond differently to immunotherapies compared to chemotherapeutic agents. The novel mechanism of action of these agents, with immune and T-cell activation, can lead to unusual patterns of response that even resemble tumour flare (pseudo-progression).^126^ As changes in tumour burden are used as a measure of response to treatment, typically using the Response Evaluation Criteria in Solid Tumours (RECIST) criteria, questions have been raised about how to assess these changes following treatment with novel immunotherapeutic agents,^127^ which has led to the development of a modified set of RECIST criteria for immune-based therapeutics (termed iRECIST).^128^ This approach allows for pseudo-progression to be taken into account when assessing initial response, but tumour measurements are still based on CT and MRI imaging methods.

Although PET-CT with ^18^F-FDG is widely used to assess the response of tumours to traditional therapy, its use is limited in assessing response to immunotherapy agents.^129,130^ This is due to the complex tumour microenvironment, in which metabolically active immune cell infiltrates can cause an expansion in tumour volume with increased glucose uptake.^130^ As the generation of an immune response leads to T-cell activation, detecting T-cell subsets (CD8^+^ and CD4^+^) within tumours might enable enhanced imaging of this response. For example, Granzyme B is a serine protease released by CD8^+^ T cells and natural killer cells during the cellular immune response, and represents one of the two dominant mechanisms by which T cells mediate cancer cell death. Early studies suggest that granzyme B might represent a biomarker for active cytotoxic immune response by PET imaging, with a potential predictive capability in immuno-oncology.^130^

### MR-linear accelerator

The MR Linac is a hybrid linear accelerator (linac) combined with an MRI scanner that can generate daily images of a patient during treatment.^108,119^ Diagnostic quality MRI yields excellent soft-tissue visualisation, and therefore offers better soft-tissue images of both the tumour and the surrounding OARs when compared to CBCT images.^22,120^ MRI also provides several imaging modalities for the identification of movement, function and physiology, which could potentially include DWI and DCE-MRI. As such, it offers scope to combine the delineation of biological indices along with standard anatomical delineation. Integrating MRI functionality with a linear accelerator could potentially make these capacities available for high precision, real-time IGRT.^120,121^ However, at present DCE is not reproducible across multiple sites, and DWI, while reproducible, is biologically non-specific.^73^ As such, these limitations would need to be overcome before these imaging biomarkers can be integrated into clinical practice.^121^

Improvements in soft-tissue imaging could allow for dose escalation to the tumour, resulting in improved tumour control. The integrated system allows for daily fast adaptive re-planning in addition to gated/tracked radiotherapy treatments, which can allow a reduction in the margins for uncertainty and tumour motion, thus decreasing the dose to normal surrounding tissue, and ultimately leading to a reduction in toxicity. Furthermore, functional imaging with MRI during a course of treatment could allow for adaptive focal boosting and personalised inhomogeneous target dosage based on response.^108^ This would involve delivering an additional dose of radiation (or ‘boost’) to areas likely to show a poor response to radiation, while keeping the dose as planned to areas showing a good response. Research is ongoing as to how best to implement this new approach in clinical care.

### Adaptive planning and artificial intelligence

To achieve real-time adaptive radiotherapy planning, including the use of the MR Linac, images need to be interpreted at the time of treatment. This involves delineation of the tumour and surrounding OARs immediately after the images have been taken, followed by registration with the treatment planning data. Once the images have been segmented, a new plan can be created. An alternative approach is to create a library of plans for each treatment course, and to then select the ‘optimal’ plan based on the current imaging. The creation of a new plan ensures that the dose from previous fractions can be taken into account in order to optimise the overall dose distribution. However, this approach can be time consuming.

The advent of auto-contouring systems integrated within the planning system offers one solution to this, by reducing the burden of re-delineating normal tissues and target volumes.^122,123^ Currently available segmentation tools include those based on image greyscale interrogation, statistical shape modelling and body atlas methods. Machine learning and integration of artificial intelligence into the planning process might also offer a solution. Machine learning has already been employed in the context of image segmentation; it can be used to automatically detect and classify different types of tissue, and can also aid segmentation by learning image context and tissue appearance and by learning appropriate priors (such as reference images or atlases) for structures and organs.^122^

### Radiomics and radiogenomics

The availability of large cohorts of images is essential to the development of auto-contouring software, and to the development of radiomics and radiogenomics, which are emerging as potential means of personalising radiotherapy. Radiomics involves the extraction of quantitative information on tumour pathophysiology from medical imaging (such as CT, MRI and/or PET), and the transformation of these data using pattern recognition tools into mineable high-dimensional data.^124^ Radiogenomics involves the integration of radiomic quantitative features (such as shape, intensity and texture, which capture tumour heterogeneity) with underlying molecular features of the tumour at the genomic level. Radiomic and radiogenomic data can be integrated with other clinical data, such as outcomes for individual patients, to develop models that might be able to predict how radiotherapy should be adapted in clinical decision making.^125^

## Conclusions

**Fig. 4:**
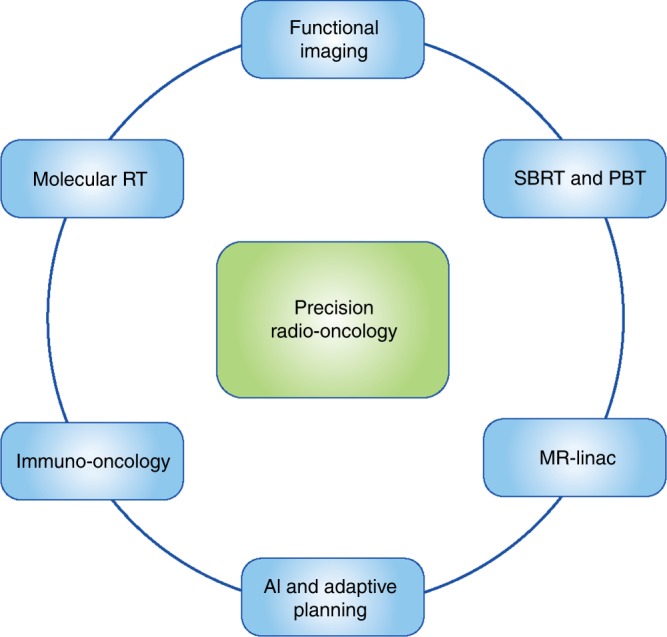
**The future of precision radiation oncology.** The future of precision radiation oncology will incorporate advanced radiotherapy techniques with functional imaging that will allow for biological dose optimisation. Novel biologically targeted radiopharmaceuticals will enable selective delivery of internal radiation, termed molecular radiotherapy. Immune modulators will be used in combination with different radiotherapy techniques in the field of immuno radio-oncology to take advantage of the synergy between these two modalities of treatment. Artificial intelligence (AI) will assist with the adaptive planning process, allowing a new radiation plan to be created, based on imaging obtained on the day of treatment. SBRT stereotactic body radiotherapy, PBT proton beam therapy, CT computerised tomography, MR Linac magnetic resonance linear accelerator

Rapid advances in imaging and radiotherapy delivery techniques have occurred over the past decade, allowing radiotherapy to be delivered at higher doses and in a more highly conformal manner. However, the future of ‘precision radiation oncology’ needs to encompass more than an ability to deliver precise technical radiotherapy (Fig. 4). It needs to incorporate new imaging biomarkers into multiple levels of treatment, including the radiotherapy planning process, allowing for biological dose optimisation, image guidance during therapy and adaptive radiotherapy. Integration of artificial intelligence into the radiotherapy planning pathway will allow adaptive radiotherapy to become a reality in clinical practice.

A particularly timely research interest is the prospect of combining precision radiotherapy with novel immune modulators. The success of novel immune modulators, such as inhibitors of cytotoxic T-lymphocyte antigen-4 (CTLA-4) and programmed death ligand-1 (PD-L1), in treating certain types of cancers, has recently led to the emergence of ‘immuno radio-oncology’ as a new field of research. Combining radiation with immune modulators is expected to lead to synergistic effects, due to the complex interaction between radiation and the immune system. However, in order to treat, and monitor response to treatment, in this new cohort of cancer patients, new imaging biomarkers will need to be developed systematically and incorporated into the radiotherapy planning process (Box 1).

## References

[1] Ringborg U, Bergqvist D, Brorsson B, Cavallin-ståhl E, Ceberg J, Einhorn N (2003). The Swedish Council on Technology Assessment in Health Care (SBU) systematic overview of radiotherapy for cancer including a prospective survey of radiotherapy practice in Sweden 2001—summary and conclusions. Acta Oncol..

[2] Sharma RA, Plummer R, Stock JK, Greenhalgh TA, Ataman O, Kelly S (2016). Clinical development of new drug-radiotherapy combinations. Nat. Rev. Clin. Oncol..

[3] Lutz ST, Jones J, Chow E (2014). Role of radiation therapy in palliative care of the patient with cancer. J. Clin. Oncol..

[4] Chow E, Zeng L, Salvo N, Dennis K, Tsao M, Lutz S (2012). Update on the systematic review of palliative radiotherapy trials for bone metastases. Clin. Oncol..

[5] Tree AC, Khoo VS, Eeles RA, Ahmed M, Dearnaley DP, Hawkins MA (2013). Stereotactic body radiotherapy for oligometastases. Lancet Oncol..

[6] Corbin KS, Hellman S, Weichselbaum RR (2013). Extracranial oligometastases: a subset of metastases curable with stereotactic radiotherapy. J. Clin. Oncol..

[7] Aitken K, Tree A, Thomas K, Nutting C, Hawkins M, Tait D (2015). Initial UK experience of stereotactic body radiotherapy for extracranial oligometastases: can we change the therapeutic paradigm?. Clin. Oncol..

[8] West CML, Huddart RA (2015). Biomarkers and imaging for precision radiotherapy. Clin. Oncol..

[9] Chang JY, Senan S, Paul MA, Mehran RJ, Louie AV, Balter P (2015). Stereotactic ablative radiotherapy versus lobectomy for operable stage I non-small-cell lung cancer: a pooled analysis of two randomised trials. Lancet Oncol..

[10] Jaffray DA (2012). Image-guided radiotherapy: from current concept to future perspectives. Nat. Rev. Clin. Oncol..

[11] Dickinson L, Ahmed HU, Allen C, Barentsz JO, Carey B, Futterer JJ (2011). Magnetic resonance imaging for the detection, localisation, and characterisation of prostate cancer: recommendations from a European consensus meeting. Eur. Urol..

[12] Yoon SH, Goo JM, Lee SM, Park CM, Seo HJ, Cheon GJ (2014). Positron emission tomography/magnetic resonance imaging evaluation of lung cancer: current status and future prospects. J. Thorac. Imaging.

[13] Wilson JM, Partridge M, Hawkins M (2014). The application of functional imaging techniques to personalise chemoradiotherapy in upper gastrointestinal malignancies. Clin. Oncol..

[14] McKibben MJ, Woods ME (2015). Preoperative imaging for staging bladder cancer. Curr. Urol. Rep..

[15] Njeh CF (2008). Tumor delineation: the weakest link in the search for accuracy in radiotherapy. J. Med. Phys..

[16] Bhide SA, Newbold KL, Harrington KJ, Nutting CM (2012). Clinical evaluation of intensity-modulated radiotherapy for head and neck cancers. Br. J. Radiol..

[17] Brock KK, Mutic S, McNutt TR, Li H, Kessler ML (2017). Use of image registration and fusion algorithms and techniques in radiotherapy: report of the AAPM Radiation Therapy Committee Task Group No. 132. Med. Phys..

[18] Castadot P, Lee JA, Parraga A, Geets X, Macq B, Gregoire V (2008). Comparison of 12 deformable registration strategies in adaptive radiation therapy for the treatment of head and neck tumors. Radiother. Oncol..

[19] Daisne JF, Duprez T, Weynand B, Lonneux M, Hamoir M, Reychler H (2004). Tumor volume in pharyngolaryngeal squamous cell carcinoma: comparison at CT, MR imaging, and FDG PET and validation with surgical specimen. Radiology.

[20] MacManus M, Nestle U, Rosenzweig KE, Carrio I, Messa C, Belohlavek O (2009). Use of PET and PET/CT for Radiation Therapy Planning: IAEA expert report 2006–2007. Radiother. Oncol..

[21] Ashamalla H, Rafla S, Parikh K, Mokhtar B, Goswami G, Kambam S (2005). The contribution of integrated PET/CT to the evolving definition of treatment volumes in radiation treatment planning in lung cancer. Int. J. Radiat. Oncol. Biol. Phys..

[22] Viswanathan AN, Dimopoulos J, Kirisits C, Berger D, Potter R (2007). Computed tomography versus magnetic resonance imaging-based contouring in cervical cancer brachytherapy: results of a prospective trial and preliminary guidelines for standardized contours. Int. J. Radiat. Oncol. Biol. Phys..

[23] Brunt JN (2010). Computed tomography-magnetic resonance image registration in radiotherapy treatment planning. Clin. Oncol..

[24] Landberg T., Chavaudra J., Dobbs J., Hanks G., Johansson K.-A., Möller T., Purdy J. (1993). Report 50. Journal of the International Commission on Radiation Units and Measurements.

[25] Landberg T., Chavaudra J., Dobbs J., Gerard J. -P., Hanks G., Horiot J. -C., Johansson K. -A., Möller T., Purdy J., Suntharalingam N., Svensson H. (1999). Report 62. Journal of the International Commission on Radiation Units and Measurements.

[26] International Commission on Radiation Units and Measurements. Report 83. *J. Int. Comm. Radiat. Units Meas.* **10**, NP-NP. 10.1093/jicru/10.1.Report83 (2010).

[27] Grégoire V, Haustermans K (2009). Functional image-guided intensity modulated radiation therapy: integration of the tumour microenvironment in treatment planning. Eur. J. Cancer.

[28] Burnet NG, Thomas SJ, Burton KE, Jefferies SJ (2004). Defining the tumour and target volumes for radiotherapy. Cancer Imaging.

[29] Ling CC, Humm J, Larson S, Amols H, Fuks Z, Leibel S (2000). Towards multidimensional radiotherapy (MD-CRT): biological imaging and biological conformality. Int. J. Radiat. Oncol. Biol. Phys..

[30] O'Connor JP, Aboagye EO, Adams JE, Aerts HJ, Barrington SF, Beer AJ (2017). Imaging biomarker roadmap for cancer studies. Nat. Rev. Clin. Oncol..

[31] Prestwich RJ, Vaidyanathan S, Scarsbrook AF (2015). Functional imaging biomarkers: potential to guide an individualised approach to radiotherapy. Clin. Oncol..

[32] Goh V, Glynne-Jones R (2014). Perfusion CT. imaging of colorectal cancer. Br. J. Radiol..

[33] Razek AA, Tawfik AM, Elsorogy LG, Soliman NY (2014). Perfusion CT. of head and neck cancer. Eur. J. Radiol..

[34] Hermans R, Meijerink M, Van den Bogaert W, Rijnders A, Weltens C, Lambin P (2003). Tumor perfusion rate determined noninvasively by dynamic computed tomography predicts outcome in head-and-neck cancer after radiotherapy. Int. J. Radiat. Oncol. Biol. Phys..

[35] Ippolito D, Querques G, Okolicsanyi S, Franzesi CT, Strazzabosco M, Sironi S (2017). Diagnostic value of dynamic contrast-enhanced CT with perfusion imaging in the quantitative assessment of tumor response to sorafenib in patients with advanced hepatocellular carcinoma: a feasibility study. Eur. J. Radiol..

[36] Hill EJ, Roberts C, Franklin JM, Enescu M, West N, MacGregor TP (2016). Clinical trial of oral nelfinavir before and during radiation therapy for advanced rectal cancer. Clin. Cancer Res..

[37] Marin D, Nelson RC, Samei E, Paulson EK, Ho LM, Boll DT (2009). Hypervascular liver tumors: low tube voltage, high tube current multidetector CT during late hepatic arterial phase for detection—initial clinical experience. Radiology.

[38] Marin D, Nelson RC, Barnhart H, Schindera ST, Ho LM, Jaffe TA (2010). Detection of pancreatic tumors, image quality, and radiation dose during the pancreatic parenchymal phase: effect of a low-tube-voltage, high-tube-current CT technique—preliminary results. Radiology.

[39] Michielsen K, Dresen R, Vanslembrouck R, De Keyzer F, Amant F, Mussen E (2017). Diagnostic value of whole body diffusion-weighted MRI compared to computed tomography for pre-operative assessment of patients suspected for ovarian cancer. Eur. J. Cancer.

[40] Yoon MY, Park J, Cho JY, Jeong CW, Ku JH, Kim HH (2017). Predicting biochemical recurrence in patients with high-risk prostate cancer using the apparent diffusion coefficient of magnetic resonance imaging. Investig. Clin. Urol..

[41] Kim S, Loevner L, Quon H, Sherman E, Weinstein G, Kilger A (2009). Diffusion-weighted magnetic resonance imaging for predicting and detecting early response to chemoradiation therapy of squamous cell carcinomas of the head and neck. Clin. Cancer Res..

[42] Albano D, Patti C, La Grutta L, Agnello F, Grassedonio E, Mule A (2016). Comparison between whole-body MRI with diffusion-weighted imaging and PET/CT in staging newly diagnosed FDG-avid lymphomas. Eur. J. Radiol..

[43] Latifoltojar A, Hall-Craggs M, Bainbridge A, Rabin N, Popat R, Rismani A (2017). Whole-body MRI quantitative biomarkers are associated significantly with treatment response in patients with newly diagnosed symptomatic multiple myeloma following bortezomib induction. Eur. Radiol..

[44] Le Bihan D, Breton E, Lallemand D, Aubin ML, Vignaud J, Laval-Jeantet M (1988). Separation of diffusion and perfusion in intravoxel incoherent motion MR imaging. Radiology.

[45] Sumi M, Nakamura T (2014). Head and neck tumours: combined MRI assessment based on IVIM and TIC analyses for the differentiation of tumors of different histological types. Eur. Radiol..

[46] Ma W, Zhang G, Ren J, Pan Q, Wen D, Zhong J (2018). Quantitative parameters of intravoxel incoherent motion diffusion weighted imaging (IVIM-DWI): potential application in predicting pathological grades of pancreatic ductal adenocarcinoma. Quant. Imaging Med. Surg..

[47] Panagiotaki E, Walker-Samuel S, Siow B, Johnson SP, Rajkumar V, Pedley RB (2014). Noninvasive quantification of solid tumor microstructure using VERDICT MRI. Cancer Res..

[48] Panagiotaki E, Chan RW, Dikaios N, Ahmed HU, O'Callaghan J, Freeman A (2015). Microstructural characterization of normal and malignant human prostate tissue with vascular, extracellular, and restricted diffusion for cytometry in tumours magnetic resonance imaging. Invest. Radiol..

[49] Bailey C, Collins DJ, Tunariu N, Orton MR, Morgan VA, Feiweier T (2018). Microstructure characterization of bone metastases from prostate cancer with diffusion MRI: preliminary findings. Front. Oncol..

[50] Dijkhoff RAP, Beets-Tan RGH, Lambregts DMJ, Beets GL, Maas M (2017). Value of DCE-MRI for staging and response evaluation in rectal cancer: a systematic review. Eur. J. Radiol..

[51] Chen BB, Shih TT (2014). DCE-MRI in hepatocellular carcinoma-clinical and therapeutic image biomarker. World J. Gastroenterol..

[52] O'Connor JPB, Rose CJ, Jackson A, Watson Y, Cheung S, Maders F (2011). DCE-MRI biomarkers of tumour heterogeneity predict CRC liver metastasis shrinkage following bevacizumab and FOLFOX-6. Br. J. Cancer.

[53] Mann RM, Balleyguier C, Baltzer PA, Bick U, Colin C, Cornford E (2015). Breast MRI: EUSOBI recommendations for women's information. Eur. Radiol..

[54] Kim, H. Variability in quantitative DCE-MRI: sources and solutions. *J. Nat. Sci*. **4**, (2018). PMC5841165 29527572

[55] Heethuis Sophie E., Goense Lucas, van Rossum Peter S. N., Borggreve Alicia S., Mook Stella, Voncken Francine E. M., Bartels-Rutten Annemarieke, Aleman Berthe M. P., van Hillegersberg Richard, Ruurda Jelle P., Meijer Gert J., Lagendijk Jan J. W., van Lier Astrid L. H. M. W. (2018). DW-MRI and DCE-MRI are of complementary value in predicting pathologic response to neoadjuvant chemoradiotherapy for esophageal cancer. Acta Oncologica.

[56] Pinker, K., Moy, L., Sutton, E. J., Mann, R. M., Weber, M., Thakur, S. B. et al. Diffusion-weighted imaging with apparent diffusion coefficient mapping for breast cancer detection as a stand-alone parameter: comparison with dynamic contrast-enhanced and multiparametric magnetic resonance imaging. *Invest. Radiol.* **53**, 587–595 (2018). 10.1097/RLI.0000000000000465 PMC6123254 29620604

[57] Baltzer PA, Dietzel M (2013). Breast lesions: diagnosis by using proton MR spectroscopy at 1.5 and 3.0 T—systematic review and meta-analysis. Radiology.

[58] Kurhanewicz J, Vigneron DB (2008). Advances in MR spectroscopy of the prostate. Magn. Reson. Imaging Clin. N. Am..

[59] Horska A, Barker PB (2010). Imaging of brain tumors: MR spectroscopy and metabolic imaging. Neuroimaging Clin. N. Am..

[60] Ogawa S, Menon RS, Tank DW, Kim SG, Merkle H, Ellermann JM (1993). Functional brain mapping by blood oxygenation level-dependent contrast magnetic resonance imaging. A comparison of signal characteristics with a biophysical model. Biophys. J..

[61] Wu GY, Suo ST, Lu Q, Zhang J, Zhu WQ, Xu JR (2015). The value of blood oxygenation level-dependent (BOLD) MR imaging in differentiation of renal solid mass and grading of renal cell carcinoma (RCC): analysis based on the largest cross-sectional area versus the entire whole tumour. PLoS ONE.

[62] Rich LJ, Seshadri M (2015). Photoacoustic imaging of vascular hemodynamics: validation with blood oxygenation level-dependent MR imaging. Radiology.

[63] Jones KM, Pollard AC, Pagel MD (2018). Clinical applications of chemical exchange saturation transfer (CEST) MRI. J. Magn. Reson. Imaging.

[64] McGowan DR, Macpherson RE, Hackett SL, Liu D, Gleeson FV, McKenna WG (2017). (18) F-fluoromisonidazole uptake in advanced stage non-small cell lung cancer: a voxel-by-voxel PET kinetics study. Med. Phys..

[65] Fraioli F, Shankar A, Hargrave D, Hyare H, Gaze MN, Groves AM (2015). 18F-fluoroethylcholine (18F-Cho) PET/MRI functional parameters in pediatric astrocytic brain tumors. Clin. Nucl. Med..

[66] Tsouana E, Stoneham S, Fersht N, Kitchen N, Gaze M, Bomanji J (2015). Evaluation of treatment response using integrated 18F-labeled choline positron emission tomography/magnetic resonance imaging in adolescents with intracranial non-germinomatous germ cell tumours. Pediatr. Blood Cancer.

[67] Huang SY, Bolch WE, Lee C, Van Brocklin HF, Pampaloni MH, Hawkins RA (2015). Patient-specific dosimetry using pretherapy [(1)(2)(4)I]m-iodobenzylguanidine ([(1)(2)(4)I]mIBG) dynamic PET/CT imaging before [(1)(3)(1)I]mIBG targeted radionuclide therapy for neuroblastoma. Mol. Imaging Biol..

[68] Chang JH, Wada M, Anderson NJ, Lim Joon D, Lee ST, Gong SJ (2013). Hypoxia-targeted radiotherapy dose painting for head and neck cancer using (18)F-FMISO PET: a biological modeling study. Acta Oncol..

[69] Thorwarth D, Eschmann SM, Paulsen F, Alber M (2007). Hypoxia dose painting by numbers: a planning study. Int. J. Radiat. Oncol. Biol. Phys..

[70] Berwouts D, Olteanu LAM, Duprez F, Vercauteren T, De Gersem W, De Neve W (2013). Three-phase adaptive dose-painting-by-numbers for head-and-neck cancer: initial results of the phase I clinical trial. Radiother. Oncol..

[71] Madani I, Duprez F, Boterberg T, Van de Wiele C, Bonte K, Deron P (2011). Maximum tolerated dose in a phase I trial on adaptive dose painting by numbers for head and neck cancer. Radiother. Oncol..

[72] Bakst RL, Lee N, Pfister DG, Zelefsky MJ, Hunt MA, Kraus DH (2011). Hypofractionated dose-painting intensity modulated radiation therapy with chemotherapy for nasopharyngeal carcinoma: a prospective trial. Int. J. Radiat. Oncol. Biol. Phys..

[73] van Elmpt W, Zegers CM, Reymen B, Even AJ, Dingemans AM, Oellers M (2016). Multiparametric imaging of patient and tumour heterogeneity in non-small-cell lung cancer: quantification of tumour hypoxia, metabolism and perfusion. Eur. J. Nucl. Med. Mol. Imaging.

[74] Taylor A, Powell ME (2004). Intensity-modulated radiotherapy—what is it?. Cancer Imaging.

[75] Alonzi R (2015). Functional radiotherapy targeting using focused dose escalation. Clin. Oncol..

[76] Chao KS, Deasy JO, Markman J, Haynie J, Perez CA, Purdy JA (2001). A prospective study of salivary function sparing in patients with head-and-neck cancers receiving intensity-modulated or three-dimensional radiation therapy: initial results. Int. J. Radiat. Oncol. Biol. Phys..

[77] Zelefsky MJ, Fuks ZVI, Hunt M, Lee HJ, Lombardi D, Ling CC (2001). High dose radiation delivered by intensity modulated conformal radiotherapy improves the outcome of localized prostate cancer. J. Urol..

[78] Kupelian PA, Thakkar VV, Khuntia D, Reddy CA, Klein EA, Mahadevan A (2005). Hypofractionated intensity-modulated radiotherapy (70 Gy at 2.5 Gy per fraction) for localized prostate cancer: long-term outcomes. Int. J. Radiat. Oncol. Biol. Phys..

[79] Bhide S, Urbano TG, Clark C, Hansen V, Adams E, Miles E (2007). cancer: a dose escalation study. Radiother. Oncol..

[80] Yu CX (1995). Intensity-modulated arc therapy with dynamic multileaf collimation: an alternative to tomotherapy. Phys. Med. Biol..

[81] Popescu CC, Olivotto IA, Beckham WA, Ansbacher W, Zavgorodni S, Shaffer R (2010). Volumetric modulated arc therapy improves dosimetry and reduces treatment time compared to conventional intensity-modulated radiotherapy for locoregional radiotherapy of left-sided breast cancer and internal mammary nodes. Int. J. Radiat. Oncol. Biol. Phys..

[82] Citrin DE (2017). Recent developments in radiotherapy. N. Engl. J. Med..

[83] Martin A, Gaya A (2010). Stereotactic body radiotherapy: a review. Clin. Oncol..

[84] Guckenberger M, Andratschke N, Alheit H, Holy R, Moustakis C, Nestle U (2014). Definition of stereotactic body radiotherapy: principles and practice for the treatment of stage I non-small cell lung cancer. Strahlenther. Onkol..

[85] Lo SS, Fakiris AJ, Chang EL, Mayr NA, Wang JZ, Papiez L (2010). Stereotactic body radiation therapy: a novel treatment modality. Nat. Rev. Clin. Oncol..

[86] Brown JM, Carlson DJ, Brenner DJ (2014). The tumor radiobiology of SRS and SBRT: are more than the 5 Rs involved?. Int. J. Radiat. Oncol. Biol. Phys..

[87] Keall PJ, Mageras GS, Balter JM, Emery RS, Forster KM, Jiang SB (2006). The management of respiratory motion in radiation oncology report of AAPM Task Group 76. Med. Phys..

[88] Amichetti M, Cianchetti M, Amelio D, Enrici RM, Minniti G (2009). Proton therapy in chordoma of the base of the skull: a systematic review. Neurosurg. Rev..

[89] Weber DC, Trofimov AV, Delaney TF, Bortfeld T (2004). A treatment planning comparison of intensity modulated photon and proton therapy for paraspinal sarcomas. Int. J. Radiat. Oncol. Biol. Phys..

[90] Paganetti H (2012). Range uncertainties in proton therapy and the role of Monte Carlo simulations. Phys. Med. Biol..

[91] Moyers MF, Miller DW (2003). Range, range modulation, and field radius requirements for proton therapy of prostate cancer. Technol. Cancer Res. Treat..

[92] Yu ZBJ, Zhang Y (2010). Impact of daily patient setup variation on proton beams passing through the couch edge. Med. Phys..

[93] Moyers MF, Miller DW, Bush DA, Slater JD (2001). Methodologies and tools for proton beam design for lung tumors. Int. J. Radiat. Oncol. Biol. Phys..

[94] Moyers MF, Sardesai M, Sun S, Miller DW (2010). Ion stopping powers and CT numbers. Med. Dosim..

[95] Yang M, Zhu XR, Park PC, Titt U, Mohan R, Virshup G (2012). Comprehensive analysis of proton range uncertainties related to patient stopping-power-ratio estimation using the stoichiometric calibration. Phys. Med. Biol..

[96] Yang M, Virshup G, Clayton J, Zhu XR, Mohan R, Dong L (2010). Theoretical variance analysis of single- and dual-energy computed tomography methods for calculating proton stopping power ratios of biological tissues. Phys. Med. Biol..

[97] Hunemohr N, Paganetti H, Greilich S, Jakel O, Seco J (2014). Tissue decomposition from dual energy CT data for MC based dose calculation in particle therapy. Med. Phys..

[98] Carhill AA, Litofsky DR, Ross DS, Jonklaas J, Cooper DS, Brierley JD (2015). Long-term outcomes following therapy in differentiated thyroid carcinoma: NTCTCS registry analysis 1987-2012. J. Clin. Endocrinol. Metab..

[99] Sohn SY, Kim HI, Kim YN, Kim TH, Kim SW, Chung JH (2018). Prognostic indicators of outcomes in patients with lung metastases from differentiated thyroid carcinoma during long-term follow-up. Clin. Endocrinol..

[100] Gaze MN, Gains JE, Walker C, Bomanji JB (2013). Optimization of molecular radiotherapy with [131I]-meta Iodobenzylguanidine for high-risk neuroblastoma. Q. J. Nucl. Med. Mol. Imaging.

[101] Strosberg J, El-Haddad G, Wolin E, Hendifar A, Yao J, Chasen B (2017). Phase 3 trial of (177)Lu-dotatate for midgut neuroendocrine tumors. N. Engl. J. Med..

[102] Wilson JS, Gains JE, Moroz V, Wheatley K, Gaze MN (2014). A systematic review of 131I-meta iodobenzylguanidine molecular radiotherapy for neuroblastoma. Eur. J. Cancer.

[103] Gaze MN (2017). Semi-quantitative scoring of skeletal metastases by (123)I-mIBG scintigraphy in high-risk neuroblastoma. Eur. J. Nucl. Med. Mol. Imaging.

[104] Gains JE, Bomanji JB, Fersht NL, Sullivan T, D'Souza D, Sullivan KP (2011). 177Lu-DOTATATE molecular radiotherapy for childhood neuroblastoma. J. Nucl. Med..

[105] Surucu M, Shah KK, Roeske JC, Choi M, Small W, Emami B (2017). Adaptive radiotherapy for head and neck cancer. Technol. Cancer Res. Treat..

[106] Bhide SA, Davies M, Burke K, McNair HA, Hansen V, Barbachano Y (2010). Weekly volume and dosimetric changes during chemoradiotherapy with intensity-modulated radiation therapy for head and neck cancer: a prospective observational study. Int. J. Radiat. Oncol. Biol. Phys..

[107] Ho KF, Marchant T, Moore C, Webster G, Rowbottom C, Penington H (2012). Monitoring dosimetric impact of weight loss with kilovoltage (kV) cone beam CT (CBCT) during parotid-sparing IMRT and concurrent chemotherapy. Int. J. Radiat. Oncol. Biol. Phys..

[108] Kerkmeijer LG, Fuller CD, Verkooijen HM, Verheij M, Choudhury A, Harrington KJ (2016). The MRI-linear accelerator consortium: evidence-based clinical introduction of an innovation in radiation oncology connecting researchers, methodology, data collection, quality assurance, and technical development. Front. Oncol..

[109] Hentschel M, Appold S, Schreiber A, Abolmaali N, Abramyuk A, Dorr W (2011). Early FDG PET at 10 or 20 Gy under chemoradiotherapy is prognostic for locoregional control and overall survival in patients with head and neck cancer. Eur. J. Nucl. Med. Mol. Imaging.

[110] Kong FM, Frey KA, Quint LE, Ten Haken RK, Hayman JA, Kessler M (2007). A pilot study of [18F]fluorodeoxyglucose positron emission tomography scans during and after radiation-based therapy in patients with non small-cell lung cancer. J. Clin. Oncol..

[111] Wieder HA, Brucher BL, Zimmermann F, Becker K, Lordick F, Beer A (2004). Time course of tumor metabolic activity during chemoradiotherapy of esophageal squamous cell carcinoma and response to treatment. J. Clin. Oncol..

[112] van Heijl M, Omloo JM, van Berge Henegouwen MI, Hoekstra OS, Boellaard R, Bossuyt PM (2011). Fluorodeoxyglucose positron emission tomography for evaluating early response during neoadjuvant chemoradiotherapy in patients with potentially curable esophageal cancer. Ann. Surg..

[113] Hoeben BA, Troost EG, Span PN, van Herpen CM, Bussink J, Oyen WJ (2013). 18F-FLT PET during radiotherapy or chemoradiotherapy in head and neck squamous cell carcinoma is an early predictor of outcome. J. Nucl. Med..

[114] Vandecaveye V, Dirix P, De Keyzer F, de Beeck KO, Vander Poorten V, Roebben I (2010). Predictive value of diffusion-weighted magnetic resonance imaging during chemoradiotherapy for head and neck squamous cell carcinoma. Eur. Radiol..

[115] Mayr NA, Wang JZ, Zhang D, Grecula JC, Lo SS, Jaroura D (2010). Longitudinal changes in tumor perfusion pattern during the radiation therapy course and its clinical impact in cervical cancer. Int. J. Radiat. Oncol. Biol. Phys..

[116] Dirix P, Vandecaveye V, De Keyzer F, Stroobants S, Hermans R, Nuyts S (2009). Dose painting in radiotherapy for head and neck squamous cell carcinoma: value of repeated functional imaging with (18)F-FDG PET, (18)F-fluoromisonidazole PET, diffusion-weighted MRI, and dynamic contrast-enhanced MRI. J. Nucl. Med..

[117] Bhatnagar P, Subesinghe M, Patel C, Prestwich R, Scarsbrook AF (2013). Functional imaging for radiation treatment planning, response assessment, and adaptive therapy in head and neck cancer. Radiographics.

[118] Moule RN, Kayani I, Prior T, Lemon C, Goodchild K, Sanghera B (2011). Adaptive 18fluoro-2-deoxyglucose positron emission tomography/computed tomography-based target volume delineation in radiotherapy planning of head and neck cancer. Clin. Oncol..

[119] Raaymakers BW, Lagendijk JJ, Overweg J, Kok JG, Raaijmakers AJ, Kerkhof EM (2009). Integrating a 1.5 T MRI scanner with a 6 MV accelerator: proof of concept. Phys. Med. Biol..

[120] Lagendijk JJ, Raaymakers BW, Raaijmakers AJ, Overweg J, Brown KJ, Kerkhof EM (2008). MRI/linac integration. Radiother. Oncol..

[121] Datta A, Aznar MC, Dubec M, Parker GJM, O'Connor JPB (2018). Delivering functional imaging on the MRI-Linac: current challenges and potential solutions. Clin. Oncol..

[122] Sharp G, Fritscher KD, Pekar V, Peroni M, Shusharina N, Veeraraghavan H (2014). Vision 20/20: perspectives on automated image segmentation for radiotherapy. Med. Phys..

[123] Jaffray DA, Das S, Jacobs PM, Jeraj R, Lambin P (2018). How advances in imaging will affect precision radiation oncology. Int. J. Radiat. Oncol. Biol. Phys..

[124] Lambin P, Leijenaar RTH, Deist TM, Peerlings J, de Jong EEC, van Timmeren J (2017). Radiomics: the bridge between medical imaging and personalized medicine. Nat. Rev. Clin. Oncol..

[125] Wu J, Tha KK, Xing L, Li R (2018). Radiomics and radiogenomics for precision radiotherapy. J. Radiat. Res..

[126] Chiou VL, Burotto M (2015). Pseudoprogression and immune-related response in solid tumors. J. Clin. Oncol..

[127] Hodi FS, Hwu WJ, Kefford R, Weber JS, Daud A, Hamid O (2016). Evaluation of immune-related response criteria and RECIST v1.1 in patients with advanced melanoma treated with pembrolizumab. J. Clin. Oncol..

[128] Seymour L, Bogaerts J, Perrone A, Ford R, Schwartz LH, Mandrekar S (2017). iRECIST: guidelines for response criteria for use in trials testing immunotherapeutics. Lancet Oncol..

[129] Juergens RA, Zukotynski KA, Singnurkar A, Snider DP, Valliant JF, Gulenchyn KY (2016). Imaging Biomarkers in Immunotherapy. Biomark. Cancer.

[130] Larimer BM, Wehrenberg-Klee E, Dubois F, Mehta A, Kalomeris T, Flaherty K (2017). Granzyme B PET imaging as a predictive biomarker of immunotherapy response. Cancer Res..

